# Justificatory explanations in machine learning: for increased transparency through documenting how key concepts drive and underpin design and engineering decisions

**DOI:** 10.1007/s00146-022-01389-z

**Published:** 2022-03-30

**Authors:** David Casacuberta, Ariel Guersenzvaig, Cristian Moyano-Fernández

**Affiliations:** 1 Philosophy Department, Universitat Autònoma de Barcelona, Barcelona, Spain; 2 ELISAVA Barcelona School of Design and Engineering UVIC-UCC, Barcelona, Spain; 3 Institute of Philosophy, CSIC, Madrid, Spain

**Keywords:** Explainability in machine learning, Justifying reasons, Decision-making, Justificatory explanations, Health

## Abstract

Given the pervasiveness of AI systems and their potential negative effects on people’s lives (especially among already marginalised groups), it becomes imperative to comprehend what goes on when an AI system generates a result, and based on what reasons, it is achieved. There are consistent technical efforts for making systems more “explainable” by reducing their opaqueness and increasing their interpretability and explainability. In this paper, we explore an alternative non-technical approach towards explainability that complement existing ones. Leaving aside technical, statistical, or data-related issues, we focus on the very conceptual underpinnings of the design decisions made by developers and other stakeholders during the lifecycle of a machine learning project. For instance, the design and development of an app to track snoring to detect possible health risks presuppose some picture or another of “health”, which is a key notion that conceptually underpins the project. We take it as a premise that these key concepts are necessarily present during design and development, albeit perhaps tacitly. We argue that by providing “justificatory explanations” about how the team understands the relevant key concepts behind its design decisions, interested parties could gain valuable insights and make better sense of the workings and outcomes of systems. Using the concept of “health”, we illustrate how a particular understanding of it might influence decisions during the design and development stages of a machine learning project, and how making this explicit by incorporating it into *ex-post* explanations might increase the explanatory and justificatory power of these explanations. We posit that a greater conceptual awareness of the key concepts that underpin design and development decisions may be beneficial to any attempt to develop explainability methods. We recommend that “justificatory explanations” are provided as technical documentation. These are declarative statements that contain at its simplest: (1) a high-level account of the understanding of the relevant key concepts a team possess related to a project’s main domain, (2) how these understandings drive decision-making during the life-cycle stages, and (3) it gives reasons (which could be implicit in the account) that the person or persons doing the explanation consider to have plausible justificatory power for the decisions that were made during the project.

## Introduction

As AI systems become all-pervasive, they increasingly have effects on the world, society, and individuals, especially affecting already marginalised people and communities (Benjamin 2019). To mitigate harms and prevent risks as they intervene in key aspects of our lives from education to law enforcement and from health to the justice system, it becomes crucial to assess how the outcomes of these systems are generated and based on what reasons. Unlike earlier AI systems based, e.g., on decision trees, modern systems, and especially those using neural networks, are said to operate as a “black box” (Holm 2019), which makes it difficult to comprehend what the algorithm exactly did and why it generated the intermediate and final outcomes it produced. There is a growing subfield within AI and AI Ethics concerned with making AI systems, especially machine learning (ML), more “explainable” by reducing their opaqueness and increasing their interpretability and explainability. This is often referred to as explainable artificial intelligence (XAI) (Barredo Arrieta et al. 2020).

In this paper, we take an alternative, non-technical yet complementary perspective towards explainability by laying our focus on the very conceptual underpinnings of the design decisions made by developers and other stakeholders during the life-cycle of a machine learning project. We focus on the role particular understandings of key, overarching concepts in a given domain play during the different stages of the machine learning process related to that domain. These understandings, in turn, and this is the core of our argument, dramatically influence other aspects of a system such as decisions about training datasets, metrics, model development, etc. These subjects frequently appear in the literature and we do not engage with them directly; we take no issue with discussions around a system’s actions or outputs neither with the math and code behind it nor with what would constitute fairness in a dataset. Rather, we concentrate on these key concepts that drive and influence decision-making.

Let us tackle an example. Consider a team working on a system (a smartphone app, for instance) that logs and tracks a person’s snoring as well as their lifestyle and other information such as heartbeat rate or weight, so the person can monitor their health and avoid complications. Many apps like these exist, and claim to use some sort of machine learning (Klaus et al. 2021).

We contend that any effort to design, develop, and deploy systems like these necessarily employs some conception or another of critical concepts related to the relevant domain as a conceptual underpinning. In the case of the app for tracking snoring, a key concept would be “health”. Furthermore, we posit that these key concepts have normative power in that they articulate and structure a myriad of design and engineering decisions all along the ML lifecycle. These concepts also prescribe standards for the domain at hand, either explicitly or implicitly. The very notion of “snoring” being a “health problem” to be monitored to prevent greater risks such as heart attacks and strokes involves a normative understanding of “snoring” as something undesirable and worth avoiding, which can be monitored thanks to the use of an app.

We take it as a premise that key concepts are thus—at least tacitly—necessarily present during design and development. Any person coming up with the idea of connecting snoring to health has some picture or another of how they are related. Any health app that tracks snoring and connects it to health presupposes this picture. Concurrently, our analysis also assumes that developers, data scientists, and others involved have a set of skills and knowledge primarily related to coding languages, technical aspects, or mathematical and statistical procedures, but are not, as a collective, well versed in the philosophy of medicine, or theories of health and well-being. (We could extend this to theories of learning, justice, happiness, and many other strategic concepts with which developers work.) Naturally, nothing prevents an individual programmer from acquiring this knowledge, but we assume that declarative knowledge about these key philosophical concepts is not a standard feature.

We will argue that a more explicit understanding of the relevant key concepts behind an AI system can be operationalized by the developers and others involved to provide rich explanations of how they are understood and how they guide design and development. These explanations might complement those provided by the system itself (which is an XAI endeavour), as well as other technical system documentation and analyses (relating to, e.g., datasets, metrics, or code). We contend that a developing team that has insights in how they understand the relevant key concepts behind their decision would be capable of offering rich explanations of their own design intentions related to the system. In turn, these explanations can enable interested parties (from team members to developers to scholars, but also from policymakers to external auditors to professionals operating a system that is already deployed) to gain valuable insights and make proper sense of the workings and outcomes of systems, possibly in connection with other data. Hayashi (2020) argues that “knowing where a [neural] network is looking within the image does not tell the user what it is doing with that part of the image”. This paper makes the case that, by fostering greater awareness of the normative key concepts behind a system, its designers could *tell* a lot, to use Hayashi’s phrasing, about what the system is doing or is supposed to be doing and what for, even if the exact details about what a system is *actually* doing remains partially unbeknownst to them.

In the next sections, we will first delve deeper into the topics of reasons and explanations (Sect. 2). Then, we will move to present different conceptual understandings of the concept of “health” (Sect. 3). In the section thereafter, we will illustrate our case and show how the different understandings of the concept of “health” might lead to different design and development decisions as well as to results that might be altogether different depending on what notion of health conceptually underpins the system (Sect. 4). In the final sections, we discuss the issues that we have raised and provide recommendations (Sect. 5). We end with a conclusion (Sect. 6).

## Reasons, explanations, and justifications

Specifically considering machine learning, explainability is roughly about making the outcomes of an AI system understandable by humans, so that it can be assessed. It seeks to break with the “black box” model in which an outcome is not accompanied by sufficient elements of analysis to explain how a system arrived at it. Being able to produce explanations has also been linked to transparency and responsibility in a moral sense (Coeckelbergh 2019: pp. 116–123). Referring to robotic systems, Wortham and Theodorou (2017) argue that AI systems should be “transparent” in the sense of enabling humans (users or other stakeholders) a general understanding of the systems’ goals and functioning. As advanced above, we do not take issue with this discussion around the computational techniques being developed in XAI to generate explanations, neither are we concerned with the discussion on whether those explanations would count as valid ones (see e.g. Coeckelbergh 2019: p. 122; Barredo Arrieta et al. 2020; Zednik 2021).

Rather, we seek to render the black box less opaque by arguing in favour of explaining the decisions made during the design and development stages of these systems. The first layer of analysis has to do with the intentionality, values, purposes, and goals embedded in them by their designers. To illustrate, when an app aims at detecting heavy snoring and recommending ways to prevent stroke or avoid marital disputes caused by being snappy due to poor sleeping patterns, it is the app’s creators who incorporate into the system those intentions and values about what is worth tracking, predicting, and recommending. Deborah Johnson (2006: p. 201) writes that the “act of designing a computer system always requires intentionality—the ability to represent, model, and act. When designers design artifacts, they poise them to behave in certain ways.”

This is not to say that this embedding of intentionality actually ensures that the system will behave in the intended manner nor that it will be used in the way planned by the designers. Science and technology (STS) scholarship consistently shows that the adoption and use of any technology is a dynamic process, which is neither fully determined by the designer nor by the artefacts’ properties, but by users, and socially and historically situated practises (see e.g. MacKenzie and Wajcman 1999).

Be that as it may, we will not discuss intentions and values either. While it is a hugely relevant topic to discuss accountability and what happens when things go South, the point to our discussion, is how goals, intentions, and even the general bird’s eye view of the functioning of a system can be traced down to *how key notions related to a particular problem domain conceptually underpin the design and development process*. We posit that explicitly explaining how a team understands these notions can facilitate an analysis of the system, the decision-making that led to it, as well as the results by providing “justificatory explanations”.

**Fig. 1:**
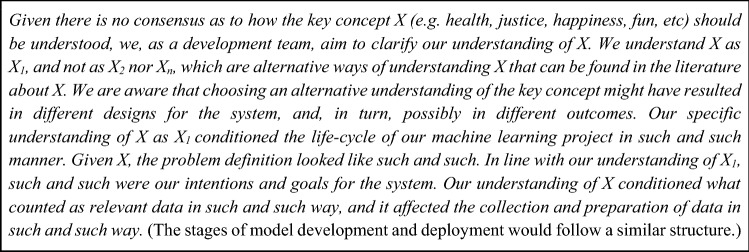
Generic example of a justificatory explanation

The term “explanation” alludes to two different but often complementary meanings. First, an explanation can be understood as an account that clarifies or details something; and second as providing reasons for an action. To integrate both meanings and avoid vagueness, we refer to “justificatory explanations”^1^ to emphasise that the explanations a design and development team generates should not only be focused on providing a descriptive account but also on providing *reasons* which could be considered to justify an outcome. Figure 1 offers a generic example of what such an explanation might look like.

Along this line, a justificatory explanation must provide not only a plausible high-level account of what has happened during the process that led to the outcome, but first and foremost, it must contain sufficient *justifying reasons* for the actions or decisions. Justifying reasons are “reasons which, very roughly, favour or justify an action, as judged by a well-informed, impartial observer” (Alvarez 2017). In other words, a justificatory explanation is adequate when it provides reasons according to which an impartial observer could accept an account as plausible and its outcome as reasonably justified.

To illustrate again, imagine we push someone out of the way at a pedestrian crossing. When asked for an explanation of what we did and why, we can say that “we pushed the person because we had to”. However, while “pushing the person” could count as a plausible account, the second, justificatory part could hardly count as an adequate justification as it fails to provide persuasive reasons to plausibly justify the action. Conversely, if we explain that we felt compelled to push someone out of the way to prevent the person from being hit by an out-of-control car, then our explanation becomes an adequate explanation as it provides both a plausible account of what went on as well as well as justifying reasons for having acted the way we did. In short, a justificatory explanation clarifies what went on and offers reasons for having acted in a particular way.

To summarise preliminarily, a justificatory explanation is, for our purposes and at its simplest, a short statement (i.e., a document) that contains: (1) a high-level account of the understanding of the relevant key concepts a team possess related to a project, (2) how these understandings drive decision-making during the life-cycle stages, and (3) it gives reasons (which could be implicit in the account) that the person or persons doing the explanation consider to have plausible justificatory power for the decisions that were made. The document serves to link the decisions made during design and development and their outcomes to a conceptual *why* that underpins the whole pursuit from the very beginning (i.e., it explains the decisions that were made in light of how key concepts were understood).

Therefore, when we talk about explainability, we are not just expecting an explanation in terms of a factual account of what a system *did* (i.e., what parameter values were set by the learning algorithm), but insights for instance on reasons *why* a system assigned those values. The question remains whether XAI can provide such explanations (Coeckelbergh 2019: pp. 121, 122). Humans, on the other hand, can and do constantly provide justificatory explanations for their behaviour.

However, any justificatory explanation may be rejected by others on the grounds of failing to provide sufficient justifying reasons (“I pushed the person out of the way because the devil made me do it”). There is a more critical caveat: an actual act may be motivated by reasons *other* than those put forward by the person doing the explanation. Furthermore, these reasons might even be fully or partially unknown to the explaining person, as it occurs with decisions that are based on intuition as is often the case with *designerly* activities (Cross 2007; Kolko 2011). Yet, that the justificatory explanation is generated *ex-post* or is not necessarily the *real* reason behind an act, need not be a critical problem for our argument. Flyvbjerg (2001) writes on the issue of “made-up reasons” that “[s]uch justification need not be illegitimate rationalisation since it can be the ex-post test of whether individual intuitive reasons are also generally valid and collectively acceptable.”

There is another caveat, that a team can produce a justificatory explanation does not mean that the decisions or acts that are accounted for are actually “justified” (i.e., containing sufficient justifying reasons, as judged by a well-informed, impartial observer). The justificatory explanation only provides some information that can enable an observer to come to an evaluative judgement. The threshold we stipulate for justificatory explanations is not that they need to be accepted as containing sufficient justifying reasons by this impartial observer, which would be desirable, but too high a threshold. After reading justificatory explanations, an observer need not agree with the development team about the rightness of the understandings of the key notions nor about the procedures nor about the quality or goodness of the decisions. The threshold to meet is that the observer can say: “I see the reasons that guided the team’s decision-making. I understand how the team conceptualised the key terms X Y Z, and I see how these concepts further influenced decisions along the line and why they did what they did.” After this, armed with this new knowledge, further evaluative assessments of the system and the system’s outcomes may be undertaken.

To exemplify and flesh out this discussion, we will consider how the design and development stages of a machine learning project are influenced by particular understandings of the concept of “health”. We will also discuss how making this understanding explicit by incorporating it into *ex-post* explanations might increase the explanatory and justificatory power of these explanations by revealing something that might be hidden, not in the opacity of the black box but on the designers’ and developers’ minds, which provides another layer of analysis for rendering the black box less opaque.

For that, let us go back to the team working on an app that logs and tracks a person’s snoring to prevent the health complications associated with it. We can safely assume that the designers believe that through tracking snoring people can become aware of potential risks and undertake actions to become more “healthy”. This belief is grounded on the knowledge that snoring can be more than just a nuisance and is related to serious health risks such as high blood pressure, heart conditions, and stroke (Yunus et al. 2018).

However, what does “healthy” mean in this context? Saying that something is “healthy” or “unhealthy” might count as making an objective description of a state. Yet, at the same time, it can also count as making an inherently normative evaluative assertion. Along this line, “healthy” is an example of a so-called *thick* concept, i.e., concepts that are both descriptive and evaluative. Thick concepts are opposed to *thin* concepts, which are either evaluative or normative. Given that thick concepts are both “action-guiding” and “guided by the world” (Williams 2006: pp. 140, 141), to say that something is “healthy” serves to guide action according to socially embedded guidelines concerning what is to be preferred and what is to be avoided, as well as what is desirable or undesirable. This is why, understanding what “healthy” means for a team is so important. If snoring is unhealthy, snoring must be avoided.

Seen from this perspective, tracking snoring makes perfect sense. Since loud snoring may be seen as indicative of a health condition, tracking snoring, and sleep *apneas*, i.e., the periods of silence when breathing stops or nearly stops, serves as a proxy for detecting many mild or severe health issues. Comparing a person’s snoring patterns to the average snoring found in healthy people might make perfect sense for detecting unhealthy patterns. We see here how considering loud snoring as a health issue is a *defining* feature during the problem-definition stage. Any claim about “loud” snoring or sleep *apneas* that are “too frequent” necessitates a measure of what type of snoring is within the normal (i.e., healthy) range. (By the way, more than five sleep *apneas* per hour of sleep is usually taken to be indicative of risk, while below that number it is perfectly fine.)

The thrust of medical deliberations is about when to attribute a particular (*thick*) concept to a biological state. Appealing to a particular conception of health, a medical professional is able to offer justifying reasons for calling a state or condition “healthy” or “diseased”. It is clear that AI systems do not have a sense of purpose and value integration in the way most people do (as when we push someone out of the way to prevent them from being hit by a car). Yet, in the architecture of any AI system, the datasets, the necessary mathematical modelling, the optimization algorithms, and other features that conform the system are put in place *for* something, i.e., to perform some tasks and achieve some goals. At its most general, this goal might be to reduce messy problems (such as determining a person’s health status) to mathematical ones that can be solved using software and numerical methods (making health-related predictions based on the normality of a person’s snoring patterns together with other data). The makers of the snoring tracking app might not go as far as to claim that a person is “diseased” based on their snoring, but if it detects risk indicators, the app might present the user with information that makes use of many *thick* notions related to health. For example, it will inform them that the snoring loudness is “above average” (which is certainly not good!), that 10 sleep *apnea*s per hour are a risk factor, and that based on the symptoms, it would be advisable for them to see a doctor for further evaluation. We see how these overarching concepts become normative and guide action.

Along these lines, the attempt to solve any problem in machine learning comes along with some notion of optimal performance in relation to a goal or solution, according to which the machine learning model is evaluated. What counts as optimal performance is thus *inextricably* connected to the way key concepts are understood. Consequently, the performance of, say, a convolutional neural network that is applied to the analysis of sounds of snoring and sleep *apneas* is assessed, for instance, in relation to the quality of the (predictive) detection of high blood pressure, heart conditions, stroke, daytime sleepiness, or motor vehicle accidents due to lack of sleep (assuming detecting these medical issues was the goal that was stipulated).

All of this is fraught with ethical issues that are related to the particular conception of health a team (medical experts, engineers, developers, data scientists, and so on) adopts, from the stage of data preparation to system deployment, either tacitly or explicitly. We contend that being aware of the particular understanding of the relevant key concepts that guide the design and development of an ML system and being able to produce declarative statements about it may greatly benefit the explainability and interpretability of a system by offering a perspective about the conceptual grounding of the system.

## A case in point: conceptions of health

We advanced above that to operate a team working on an AI system related to health, the team members necessarily must have at least some picture or another of the concept of “health”. But what is “health”?

At first sight, “health” appears to be, at least for those of us in the Western world, an unambiguous notion having to do with not being “sick” or “ill”, and perhaps with doing and feeling well. For the purposes of this paper, we call this informal approximation to the concept an “everyday understanding”, which is the primary layer to structure ideas and evaluations. In the case of health, an everyday understanding articulates ideas about how the body functions and should function, and what functionings are especially relevant and worthy of further consideration. Furthermore, the way we understand health is interrelated to how we conceptualise the human body (for example, as a deterministic machine that breaks down with disease or as an organism that is part of a dynamic system involving the physical but also the social). These everyday understandings of health and other concepts have theoretical underpinnings arising from the wider scientific and non-scientific theories, cultural traditions, and folk knowledge (Trollope-Kumar and Last 2002: pp. 685, 686).^2^

Without aiming to exhaust the topic, in this section, we will review three influential positions about health that in one way or another might underpin our everyday understanding of health. The discussion will allow us to observe that “health” is far from an unambiguous and self-evident notion. We will consider one “naturalist” theory of health in Sect. 3.1, whereby “health” is a notion that is free of normative values and determined by empirical facts. In Sect. 3.2, we will also consider two “normativist” theories, whereby the notion of “health” necessarily reflects value judgments.

### A naturalistic theory of health: Boorse’s “biostatistical” theory

Possibly, the most vigorously debated naturalist theory of health is the “biostatistical theory” of health proposed by Christopher Boorse (1977, 2014). The theory aims to provide a completely objective account of health and disease. In Boorse’s empirical view, health and disease are nothing else than biological states. To the naturalists, to say something is healthy is merely to give a value-neutral description of an empirical fact. In this way, the naturalist account aims to give a value-free, objective definition of what health and disease are. In the words of Boorse (1977: p. 543): “if diseases are deviations from the species biological design, their recognition is a matter of natural science, not evaluative decision”.

For Boorse (1977: p. 542), health is “normal functioning” and diseases (or “pathological conditions”) are “internal states that depress a functional ability below species-typical levels” relative to sex and age. (Boorse 1977: p. 542, 2014: p. 684). Health is to be understood as the “total absence of pathological conditions” (pp. 683–684).

In this theory, typical levels for a species are those close to the statistical mean (Boorse 1977: pp. 558, 559). To determine whether an organism is healthy in relation to the species-typical level Boorse introduces the notion of “reference class”, “a natural class of organisms of uniform functional design; specifically, an age group of a sex of a species” (Boorse 1977: p. 555). For example, according to this view, to determine if a person has normal levels of testosterone, we must first determine this person’s age and sex and use this as a reference class, as there is much statistical variation regarding testosterone levels among males and females and across the age range. Since species design seems to be contingent on sex, age, and, in some cases, race, the statistical abstractions should be made from reference classes smaller than species (Boorse 1977: p. 558), e.g., “a 35 years old white woman”.

For Boorse (1977: p. 554), “the normal is the natural”, an organism is healthy when its functioning conforms to its natural design. What is normal functioning, then? Again, normality must be understood in a statistical sense and function in a biological sense. “A normal function of a part or process within members of the reference class is a statistically typical contribution by it to their individual survival and reproduction” (Boorse 1977: p. 555). Typical contributions are those “within or above some chosen central region of their population distribution” (Boorse 1977: p. 559). This entails that abnormal functioning is not a sufficient condition by itself to be regarded as a disease. To count as such, the functioning has to be subnormal, i.e., “below” average functioning and thus detrimental in some way to the highest level goals of survival and reproduction.

According to biostatistical theory, health “in a member of the reference class is normal functional ability: the readiness of each internal part to perform all its normal functions on typical occasions with at least typical efficiency” (Boorse 2014: p. 684). The theory concedes that there are dysfunctional states that are nonetheless statistically normal, such as tooth decay. However, since these conditions are due to environmental agents and not are in the design of the species, they do not contradict the important role statistical normality plays in the theory; they cannot be considered functional designs that are shown to be empirically typical (Boorse 1977: pp. 555, 556, 2014: p. 705).

#### The problem with reference classes

It is clear that to assess the normality of a biological state, we need some sort of benchmark of normality, a reference against which things are compared. “Normal” snoring can be determined statistically, but how can we determine levels above which snoring becomes “unhealthy” or risky? While Boorse convincingly shows that comparisons at a species level can be inoperative and that a smaller reference class is needed. It is not clear why it is appropriate to factor sex, age, and race instead of other criteria. Can we possibly find other suitable classes? Building on Kingma (2010), it could be argued that there are no empirical facts that determine that “young adults” is an appropriate reference class, but “people with obesity” (who are more prone to snoring) or “people with tooth decay” are not.

As Moore (1993) famously insisted, we cannot base a normative assertion on a natural property, and as the case of tooth decay being statistically normal indicates, more seems to be required for something to be “healthy” than to say that it is “normal” in a statistical sense. Furthermore, if Boorse’s account is to be value-free, what constitutes an appropriate reference class and what does not should be justified without normative judgments. However, we would reject “smokers over 65 with obesity” or “young adult males with tooth decay” as suitable reference classes, because they conflict with our most basic intuitions about being healthy. We do not see them as appropriate reference classes, because smoking, tooth decay, and obesity are clearly something to be avoided from a medical perspective. However, this rejection *is* a normative choice, it is not value-free, it reflects our cultural, political, social, aesthetical, and even, perhaps, religious values.

The biostatistical theory does have many virtues, “once the reference classes are fixed [it] gives an accurate and value-free analysis of health and disease” (Kingma 2010: p. 132). However, it necessitates auxiliary normative elements (for instance, for the fixation of the necessary reference classes), which strongly underscore the need to declare them if one wants to make a system explainable, as these values that drive analysis cannot be inferred from the dataset.

### Two normativist characterizations of health

Normativists, contrary to naturalists, argue that defining health inevitably involves all sorts of norms and values. Therefore, to say that something is healthy is to irremediably give an evaluation. While normativism does not necessarily imply a full-blown socially constructed view of all biological processes or states, normativists generally argue that human values, interests, and beliefs are determinants in the identification of the relevant processes. This value-ladenness is thus inextricably linked to the notion of health. Analogously, the related notion of “disease” is not objective in the same way the mineral composition of a rock can be said to be objective—there is no “natural, objectively definable set of human malfunctions that cause disease” (Murphy 2021).

The general assumption of the normativist approach is that any conception of health, illness, and medical needs should not be reduced to the biological factors. On the contrary, it is necessary to adhere to a broader, holistic, and relational concept of health. Phenomena such as chronic stress, a product of the enormous changes in the environment in which we live, show the inefficiency of a biological medical model focused on bodily symptoms, which neglects to address the links of diseases with the environment and psychosocial and productive factors (Marmot and Wilkinson 1999; Wilkinson and Pickett 2006). Thereby, the emergence of perspectives based on consideration of social determinants of health has become widespread in the last years.

In contrast to Boorse’s position, normativists argue that medical diagnosis does not consist of descriptions of empirical data, but that such descriptions always contain subjective, cultural, or ethical evaluations. According to the normativists, quantitative or statistical data are not sufficient to determine the health status of a person, but rather a holistic assessment of the general state of the person is required (Nordenfelt 2007: p. 6). Lennart Nordenfelt, one of the main representatives of normativism, argues that health is not identified with the absence of illness, but refers to the ability to achieve (non-arbitrary) vital goals (Nordenfelt et al. 2001; Nordenfelt 2007). This conception of health implies accepting the intertwining of values conditioned by a subjective agent, its goals, and the circumstances in which the agent acts. Similarly, an illness is not an anatomical defect of the “normal body”, as in Boorse’s theory, but a situation perceived as problematic. The subjective recognition of a situation or state as problematic would thus constitute the fundamental prerequisite for seeking medical help.

#### Health according to the World Health Organization (WHO)

The definition proposed by the WHO has been characterised as “idealistic” and to “give no help” (Bircher 2005). By defining health as “a state of complete physical, mental and social well-being and not merely the absence of disease or infirmity” (WHO 1948), health seems to become an unreasonably high standard. Such conception of health is incomplete and ambiguous, and has been criticised as postponing the difficult work of understanding what it is. Understanding health as a maximum state of well-being that cannot be reduced to biological aspects, the WHO’s approach is also far from Boorse’s naturalism. However, its ambitious definition hardly helps to guide medical applicability.

Furthermore, the term “state” used by the WHO definition of health refers to an absolute, static, and decontextualized moment, which does not take into account the changing and variable conditions of people. Presenting health in such an ambitious way makes it difficult for it to be durable over time and compatible with minor discomfort. Such a definition obscures how to prioritise health care or treatment, because the number of unhealthy people can be constant and endless. For this reason, other normativist approaches have chosen to understand the “health-illness process” as a dynamic experience involving both dimensions, shaped by personal and group history. Thus, instead of being presented antagonistically to illness, health would reflect a continuous process of changes between complex moments in life, during which favourable and unfavourable conditions may coexist. In line with this dynamic and holistic definition of health, the capabilities approach provides a meaningful contribution.

#### A *capabilitarian* approach to health

The theory of health advocated from the capabilities approach is not value-free and anchored in biology, neither is it one that is wholly centred on the concept of disease. Instead, the conception of health is understood as the person’s true, actual ability to achieve or exercise a cluster of basic human activities (“functionings”). These activities are in turn specified through reasoning about what constitutes a minimal conception of a life with human dignity in the modern world (Venkatapuram 2011: pp. 42, 43).

The capabilitarian perspective to health may be reminiscent of Nordenfelt’s postulates, which defines health as the ability to achieve vital goals. Both are committed to focusing on the abilities and not the actual achievements or functionings, but Nordenfelt’s arguments have a significant drawback: a lack of substantive content to describe what these putative vital goals might look like. However, a concept of health should be much more explicit about these vital goals to help us determine an adequate environment to flourish and be healthy. A capabilitarian approach to health can be taken to overcome the challenges encountered by Nordenfelt’s theory in regards to the lack of substantive content. Martha Nussbaum has provided a list of central capabilities, which, though hotly contested by other capabilitarian scholars, enumerates the minimal capabilities (e.g., life, bodily integrity, affiliation, etc.) that are indispensable for a life worthy of human dignity (Nussbaum 2006: pp. 76, 77).

A meaningful advantage of the capabilities approach is the idea of a sufficient threshold (Nussbaum 2006). Unlike some other normativist perspectives as Nordenfelt’s, the inclusion of a minimum level of capabilities or vital goals helps to rethink the individual bonds between the pluralistic and subjective experience of health and a common basis for being healthy. As Thomas Schramme (2007) has noted, the definition of vital goals proposed from normativism may be too broad. It is not completely reasonable that a person’s subjective preferences can determine their health status. Schramme (2007: p. 14) uses the example of an ambitious athlete to illustrate this objection. For this athlete, it could be a great disgrace not to have professional success, but it would seem odd to argue that they have become unhealthy. Although they may feel unhappy, there is still a gap until they can be diagnosed as unhealthy. Certainly, there should be a spectrum or a sliding scale from complete health to maximal illness, but here the use of thresholds becomes crucial. A list of basic capabilities whether participatively defined by a community or established by a scholar (such as Nussbaum’s list) constrains the scope and adds a degree of intersubjectivity by adding sufficiency levels (i.e., a minimal threshold).

The upshot of all this is that if a developer or designer has a normativist conception of health (however, tacit), this divergence will lead to different decision-making regarding the promotion of health than a developer who holds a biostatistical, naturalist conception of health. A team working from a normativist approach might focus more on working with individual and collective goals, and subjective states than purely on parsing data from medical statistics. Yet, this divergence cannot easily be inferred from a dataset or a model.

## The role of our conception of health when building algorithms

**Fig. 2:**
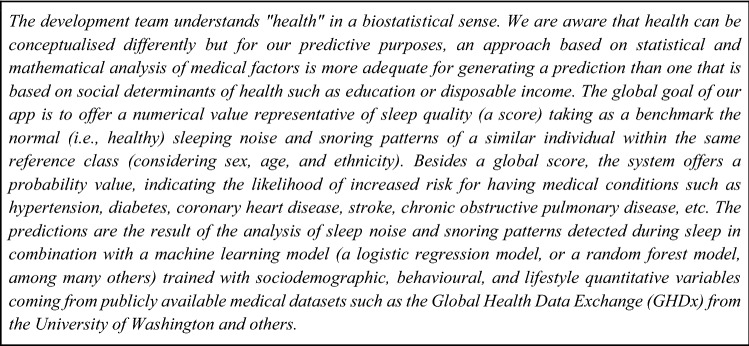
Example of a justificatory explanation for an app to detect and evaluate snoring

In this section, we explore how fairness, explainability, and other ethical concerns may shift depending on the implicit or explicit concept of health guiding our model. The definition of health that the system developers and designers are going to use may have an impact in different parts of the lifecycle of a machine learning algorithm. Because we take a non-technical approach to this paper, we propose a simplified view of the different stages conforming the lifecycle: (1) problem definition, (2) data collection and preparation, (3) model development, and (4) model deployment (see e.g. Ashmore, et al. 2021; Google 2017). We will explore some of the effects that different conceptions of key notions might have during these stages and how every stage may be conditioned by these alternative understandings. As a starting point, Fig. 2 illustrates the justificatory explanations around the concept of health that could be produced by a team. In this case, their understanding is grounded on a naturalist perspective.

### Problem definition stage

When defining which variables will be relevant for our model, the concept of health that guides our reasoning may have implications. For example, from a perspective of privacy, a naturalistic approach is more suitable to keep sensitive data safe. A machine learning model that is based on a Boorsean approach may need less sensitive information of users than a capabilitarian one, which implies collecting information about lifestyles, emotional well-being, vital goals, and life plans which are highly sensitive. Therefore, a model that uses a naturalistic understanding of what health is becomes more privacy-friendly (Cavoukian 2009).

However, when considering explanations, normativity should be added to the equation. As we stated above, an adequate explanation needs to justify the outcome. Explanations provide us with reasons for having acted or acting in a certain way, offering us more than just some correlations between numbers. Causes and conditions do play a role in the explanation, but the reason for a certain course of action is always needed if we are looking for an explanation (Von Wright 1981).

Open approaches like the one developed by the WHO turn unnecessarily difficult to present a coherent explanation of what the algorithm ultimately does. However, because it is the official characterisation by the reference world organisation about health, it seems likely that developers and researchers would quote this definition, only to find out that it is useless on its own for developing a machine learning model. We imagine that researchers faced with these difficulties would decide to use data proxies instead that may capture in theory what health is according to the WHO.

It is especially at this stage (in relation to the deployment stage) where we believe that our proposal can be particularly beneficial as it aims to shed light on the fundamental assumptions that are made by a team. For instance, as we will see below, these assumptions can drive the use of simplistic proxies to represent complex realities in other project stages, which can lead to unacceptable outcomes. Moreover, conceptual clarity during this first stage affords a broad perspective to the prevention of risks and harms that goes beyond technical fairness metrics that could be applied at later stages, such as comparing and evaluating error rates per group to detect inadmissibly discriminatory outcomes (see, e.g., the case of COMPAS famously analysed by Angwin et al. 2016).

By exploring key concepts and their entanglements with design decisions through justificatory explanations all project stakeholders (including civil society, administrators, and policy makers) can reflect on and evaluate the very framing of the problem to detect problematic aspects that might otherwise fail to be captured by analysing the datasets or the code from a mathematical or statistical perspective. An explanatory justification (or the lack thereof!) may, for instance, serve to examine the scientific validity of the assumptions that underpin a system.

To illustrate, imagine a team working on a video surveillance system that aims to “ensure safety and security” in public spaces by, for instance, focusing on the prevention and reduction of crime through real-time video analysis. A justificatory explanation would require the team to clarify these concepts (safety, security, crime, prevention, etc.). It would also mandate to explain how the captured video data will conceptually map to the stipulated goals and to the key terms (for example by saying that the task will be to generate a prediction of criminal intention through analysing location, gait, and facial data of individuals). When the justificatory explanation is reviewed by a third party, questions could possibly arise about what kind of model of criminal behaviour connects gait to criminal intentions. These questions might lead to further probing the concepts and the very soundness of the theories (or lack thereof) behind the project. This, in turn, might serve to gain insights on potential risks and harms and to challenge or reject the project as a whole.

### Data collection and preparation stage

We should stop considering data collection a safe, objective procedure. As shown by Gebru et al. (2021), a vast array of scholars’ data plays a critical role when developing an ML application. A model needs to match training and evaluation datasets with the reality it tries to model. Inaccuracies and misrepresentations or underrepresentation of segments of the population may lead to mistakes, biases, and unjust impacts, i.e., discriminations.

The conception of health that underpins an ML project will have relevant effects on the data collection and preparation process. As explained in Sect. 4.1 , choosing a Boorsean approach may imply the need for fewer variables in the problem definition section. Similarly, the Boorsean approach can be developed by accessing fewer data sources than a capabilitarian one, which could be assumed to be more difficult to obtain. As a matter of fact, an algorithm created using a naturalistic view of health needs just biological data, which are generally included in the patient’s medical records and medical databases. Therefore, one or a few sources should normally suffice. Instead, if we are considering a capabilitarian approach, many other data sources will have to be included, possibly something coming from digital social networks in which users share lifestyles and feelings or other forms of user-generated data. As stated in seminal studies like Sweeney (2000) and more recent ones like Cecaj et al. (2016), or Yin et al. (2015) in supposedly anonymized databases, individuals can be re-identified when crossing references between different databases.

When considering health disparities and biases, the most common relevant variables are race, sex, gender, age, and socio-economic status (Underwood et al. 2012; Kawachi et al. 2005; D'Ignazio and Klein 2020; Criado Perez 2019). The first variables are quite manageable from a data science point of view, but socio-economic status is more difficult to calculate and obtain. A common proxy, i.e., a value that can be seen as representative of another value, for socio-economic status is insurance type and has been used in several studies such as Seyyed-Kalantari et al. (2020) or Chen et al. (2019). Geographic data (Soobader et al. 2001), property value (Ware 2019), or even online activity in social networks (Levy Abitbol et al. 2019) have been used as proxies, as well. In many countries, those types of data can be retrieved with ease from public databases and turned into numbers for the model. However, neither insurance type, location, property, nor social networking activity are equivalent to socio-economic status, and socio-economic status does not cover by itself all the nuances of a normativist approach to health. Moreover, depending on the proxy we use to capture “socio-economic status”, we will consider some minority subgroups in our metrics but not others. Using social networking data—a trending fad right now—will not take into consideration people within the digital divide that does not have access to digital media. Other proxies like property value or geographic data will not be able to capture all the richness included in Nussbaum’s list, for example, not considering people that may have basic needs covered, but do not fare well in a mental well-being score, nor the relative value of material resources (a person might own a bike, which might be worthless without cycling paths around).

From a naturalist perspective, weight and age might be relevant and decisive factors in the snoring app to keep track of and process, as a person that is overweight or has obesity is statistically more prone to snoring. Conversely, from a normativist perspective, the issue of where the person was born, lives, and works might be more relevant. According to the “Social Determinants of Health” perspective, it is a truism to say that having poor access to healthy dietary options, good housing, or opportunities to exercise can also be linked to heart disease or stroke. Also from this perspective, if one wants to contribute to improving a person’s health, it might make much more sense to track these issues than to be concerned with snoring and noise produced during sleep.

### Model development stage

As we have argued in Sect. 3.1, a naturalistic approach cannot capture all the nuances we associate with our conception of health. Calling someone healthy is not value-free, and it is something more than just being free of disease. In an actual ML development, we possibly need to include some normativist elements in our definition of health. But that is easier said than done. Despite trying to present a more detailed and specific definition of the different dimensions of a healthy human being, the capabilitarian approach is not fully articulated yet to serve as a proper normative framework for health. As we have mentioned in Sect. 3.2.2, Nussbaum (2006: pp. 76, 77) gives us a list of central capabilities that minimally ensure human flourishing. Some of those characteristics, like “length of lifespan”, can be easily converted into numbers and included in a machine learning algorithm, but some others like “possess practical reason to form a conception of the good” or “be able to play” are difficult to define in non-ambiguous terms, and once defined, it is not easy to turn them into a set of numbers that make sense nor to find representative data sources or adequate proxies.

Nussbaum’s presentation of health is good enough for philosophical and ethical purposes, but is not specific enough to give a coherent background that can be translated into a self-explanatory machine learning model. Therefore, a new type of effort is needed, interdisciplinary research in which ethicists, philosophers, and data scientists work together to generate normativist understandings of health with much more detail than current ones. In the case of the capabilities approach, for instance, this would be about clearly defining the relationship between a capability and a legitimate data proxy.

No matter how well we define our capabilities, some biological data will need to be included, as well. And not merely because of the obvious biomedical reasons. Those data have to be included if we want a system that is both accurate and just. As we mentioned before, the main biases found in algorithms for health are related to age, sex, gender, race, and socio-economic status. When a group becomes a majority, underrepresented subgroups may become vulnerable to being mistreated when deploying the model. Such vulnerability is not easily solved just by increasing the number of patients that are members of such subgroups. In Seyyed-Kalantari et al. (2020), large public datasets currently used to generate machine learning algorithms to diagnose chest X-rays are analysed. Authors observe that female patients were the least favoured subgroup, even though the proportion of female patients was only slightly less than male patients in the different datasets analysed (Seyyed-Kalantari et al. 2020: p. 239).

### Model deployment stage

When and why will our ML model be used? And who are the subjects to whom the model will be helpful? To decide when it is helpful to deploy the model and use it on the real population, we need medical and health criteria to do so. That means that our conception of what health is will play a role when deciding whether we need to use a specific machine learning model with a given population or not.

Deploying a model targeted to a specific population that we think have special issues may become a self-fulfilling prophecy. Depending on the results of the algorithm, a specific medical procedure may be used in such a population. Then, the data will be used to improve the algorithm in another lifecycle, producing a feedback loop that builds and amplifies those specific medical needs of such a segment of the population.

The phenomenon we are postulating here is akin to the well-known self-fulfilling prophecy of turning neighbourhoods less safe by monitoring them with video cameras. Let us suppose that the city police considers that one neighbourhood does have more criminal activities than others. Therefore, they deploy surveillance cameras there to detect criminal activities. Because there are more cameras, it is easier to spot criminal activities, so more crime is detected and there are more arrests in this neighbourhood than in others. Data on arrests and convictions are included in future models, so there is more “evidence” that the surveilled neighbourhood is less safe. Naturally, there may be other neighbourhoods as dangerous as this one, or even more, but because they are less monitored, such data are below the radar. The decision to deploy cameras is never value-free, but the values that drive the deployment may be perversely hidden in the data that is generated and further used (see e.g. Alexander 2019).

Likewise, if a model is deployed with a Boorsean perspective of health in mind, it will give a false impression of more precision as it gathers more data (e.g., an app for tracking snoring aimed at people with obesity, will likely *work* in predicting potential strokes or diabetes). However as it limits the relevance of social indicators of health, it may leave out sections of the population with health issues that can be understood through a capabilitarian approach to health but are not well represented in a purely statistical approach like the Boorsean (if we focus primarily on evaluating snoring we pay less attention to other social factors that might be much more relevant such as education or the availability of a health insurance plan). Moreover, under a naturalist conception of health, a “healthy” baby may be one within statistical normality, whereas a baby with Spina bifida, Down syndrome, or any disability for that matter, may be counted as “unhealthy” due to being statistically rare. However, these classifications are dramatically fraught and linked to the balances of power and tensions of the present and the past.

Instead, if using a capabilitarian approach, a model based on quantitative predictions will not be helpful, and we would need a more participatory approach instead, in which citizens can democratically decide which health issues are more relevant and which sections of the population are more in need of study, analysis and deployment, possibly in relation to capabilities that are worth promoting. It is a truism to state that the postal code is at the group level a better predictor of health and well-being than many medical variables combined.

In this stage, ML models can and are constantly optimised. Further model iteration, interpretation, and evaluation of results are critical. For example, if not developed with inclusivity in mind, ML could exacerbate health care disparities in dermatological care (Adamson and Smith 2018). A purely naturalist approach might overlook this need by laying the focus on statistically normality and by assuming that health is an objective matter.

## Discussion

When we develop an algorithm to track and predict health issues in humans, disparities will inevitably show up. To mitigate potential harms, the first step we need to consider is where such disparities come from, and whether they should also be considered discriminatory and conducive to unjust impacts.

To do so, we need to consider all the phases in a machine learning model development, but especially the first ones, problem definition and the data collection and preparation stages. Here, problems are framed in a particular way and people and their relative health are reduced to data put into a set of rows and columns on a CSV file. We posit that problems are “framed” often in subjective and dynamic ways, which depend on particular conceptions of the concepts associated with a domain, as well as on the pursued outcomes, power dynamics, and many other historical contingencies. We should infuse meaning beyond the numerical values that make up the datasets, so we know what those numbers stand for and what are the aims behind them. We will not be able to characterise such aims unless we understand our ultimate goal: in the case of the app for tracking snoring, it was the promotion of health. But still, as we have seen, health is an elusive notion that can be conceptualised in multiple ways. Therefore, we need to have a proper understanding of what actual concept of health has led to a given system. For that, we need to clarify what we actually talk about when we talk about a system that contributes to improving health or assists users with health-related decisions.

At the same time, we cannot just import a list of basic human capabilities as those defined by Nussbaum (2006: pp. 76, 77) into a system and expect our algorithm to generalise and predict anything useful from that. Proxies might be unavoidable. Yet, it is crucial that selecting a proxy is guided by the developers’ mind set. In other words, to generate explanations that are both accurate and relevant for humans, we need to understand clearly how each element in our understanding of health (or any other relevant key concept) relates to specific proxies, and what are the limitations of such links. Even non-medical data might turn out to be relevant for health-related endeavours, depending on our understanding of the issue. Data about a person’s engagement in community activities or their early childhood education might be a better indicator of health risks than their heartbeat rate or the noises produced during sleeping. But again, to come to this realisation, a developer needs to be guided by a particular conception of health.

We should also take into consideration cultural differences. As we stated in Sect. 3.2.2, capabilitarian models are open to cultural differences. That is good for a general, philosophical model, but the explanatory justifications for our models should take into account the rapidly changing nature of cultural identification, and update the model’s premises to the specific cultural background we want to reflect. We now label it as neurodiversity or as mere sexual orientation what a couple of decades ago was commonly considered a disease (or even a crime). Today, many more people identify themselves as “Black” or “Latino” than 3 decades ago. Furthermore, some people are considered “White” in one continent and “Non-white” in another. Even our envisioning of sex as something purely biological is rapidly changing as new understandings of gender arise. We should be extremely careful with older datasets, which may no longer reflect the values and identities of the society we are trying to model.

A map is not the territory, and the model is not the thing we are modelling. Furthermore, artificial intelligence applications are sometimes presented as having a real understanding of the things mapped, when it is not so. If we remove any sort of social determinant of health in our model, we will not be able to consider how socio-economic status, race, or gender leads to differences in development and on the way people are treated when entering the health system. A machine learning model primarily based on a naturalistic approach to health will that way become a very simplistic map with no chance of giving us an accurate picture of how health and sickness are not equally distributed among a population. It might also occlude the discovery of the reasons for such inequality. Without an explicit declaration of what is understood behind a system’s key concepts (in this case health), many issues may remain hidden and negatively impact the lives of those affected by the system, both in the present and in the future.

Taking the map as the territory is also problematic for the general public, which has access to the world of artificial intelligence mainly through generalist media and social networks, and tend to believe that machine learning algorithms do really “understand” faces, diseases or human behaviour in a way akin to how a person does.

When developing a machine learning algorithm for health purposes, we will measure some target variable that is going to be a construct. We should see the predictions of the model as an indicator of some complex combination of properties in the individual and not as real property in itself. When that app that tracks snoring tells us that someone’s sleep score last night was, say, 75 due to heavy snoring, neither the development team nor the person snoring nor the medical staff should take such a number at face value. The score value calls for a hermeneutical effort, as it needs to be interpreted to discover the meaning behind the number and how it links to a general understanding of what health is.

A simile may be useful here. Consider crime risk assessments generated by machine learning algorithms. Governments, the press, and software vendors and manufacturers present such models as predicting how crime will rise or drop depending on the neighbourhood. However, that is not really what the algorithm does, as the database does not include the “number of crimes detected” but the “number of crimes previously solved by the police and judged in a trial”. And those numbers may be very different. If the police tend to make arrests in some neighbourhoods but less in others, we will not be measuring crime, but police biases. If some judges are more strict than others, we will not be really measuring crime again, but judicial biases (Yapo and Weiss 2018; Lee 2018). Again, just like with “health”, we see here how a clear understanding of the concept of “crime” is crucial for the discussion. Consider how these biases are actually happening, despite the link between crime and arrests being quite close, and how things can possibly go wrong when we use an individual’s “net worth” or “activity on Twitter” as a proxy measure for health.

Another important lesson here is how we should do away with having accuracy as the only factor in the creation of a model and to consider the inclusion of other metrics in search for fairness, such as equal accuracy across groups (Srivastava et al. 2019). A normativist conception of health would sway a developing team in that direction. In the same way, adding mathematical elements to protect the patient’s privacy, such as differential privacy (Dwork and Roth 2014), will make the model somewhat less accurate. This should not be a problem. We are not considering abstract mathematical functions but models that will be used to classify humans and a little less accuracy is welcomed if we get more equity and a better understanding of the real relationship between those scores and the real lives of people and their health.

Moreover, a naturalistic model of health is inherently more prone than a normativist to suggest that accuracy is possible at all. We can measure heart rate accurately and derive a benchmark from these measurements, but from a normativist perspective, it makes no sense to talk about an accurate prediction of affiliation, the capacity to feel love and be loved, or bodily integrity. Yet, all of these themes can be linked to health issues.

So far, we have focused on the design and development of a whole system (a smartphone app that tracks snoring) to exemplify our discussion, but our arguments in favour of transparency about concepts are also valid for parts of the process. The need to declare how a team understands health becomes especially relevant for example when pretrained third-party models are integrated into other systems via APIs, as it often occurs. Conceivably, the third-party model could have been trained by people with different conceptual underpinnings. Without conceptual transparency, disaster is served.

As it becomes clear from Figs. 1 and 2, our proposal is rather modest in technical terms. One possibility would simply be to add metadata to the code. This could be done in the way the problem type is declared in a model’s metadata (e.g., “Problem type: summarization”) or like the environmental impact of model training expressed in CO^2^ emissions is shared in the metadata by the Hugging Face community (Hugginface 2021). It could also be operationalized in terms as simple as adding a text section in the system’s documentation where the key concepts for the system are considered.

Another possibility would be to integrate these justificatory explanations into the “model cards” framework proposed by Margaret Mitchell and her associates (Mitchell et al. 2019). This framework encourages transparent model reporting and documentation accompanying trained machine learning models. Model cards include information about model details, intended use, metrics, evaluation, training data, etc. While there is certainly much common ground between our proposal and the model cards framework, the focus of our paper is much narrower and goes one step further specifically in what the authors refer to as the area of “ethical considerations” where they recommend to consider questions such as “Is the model intended to inform decisions about matters central to human life or flourishing—e.g., health or safety? Or could it be used in such a way?” (Mitchell et al. 2019: p. 6). Likewise, another possibility would be to include the explanations into the “datasheets for datasets” proposed by Gebru and her associates (2021), where the motivation of a dataset, its composition, collection process, recommended uses, and so on are documented. Gebru’s framework suggests including information about the “reasons for creating the dataset”, “for what purpose the dataset was created”, and “if there were specific tasks in mind?”.

Despite the many similarities between our proposal and Mitchell’s and Gebru’s more comprehensive frameworks, ours underscores the need to state the way the key overarching concepts are conceptualised. We have argued that it is not enough to talk about “flourishing”, “health”, or “safety”, because these notions are intrinsically fraught concepts that need further clarification to truly offer the transparency and the explanatory justifications everyone expects. The same can be said about purposes or tasks: saying that the purpose was to improve health will not cut it.

## Conclusion

As Gebru et al. (2021) show, the way datasets are defined is critical to assure a useful and ethical behaviour when using a model in the wild. In this paper, we have shown that, when creating AI systems, our conception of health may have implications for all the stages along the lifecycle. Using a Boorsean approach to health facilitates the generation of simpler and more accurate models, but we may generate simplifications and ethically inadmissible discriminations in the process by excluding people outside the normal distribution or by choosing biased reference classes. A system based on that view might require reference classes that appear to be objective but are not only highly contested but also dependent on norms and values (e.g., race and sex). By being grounded on a broader understanding of what health is, a capabilitarian approach will possibly lead to more complex and less -accurate algorithms, but ones that are *prima facie* less prone to being biased and causing negative impacts.

In short, if we want to create machine learning models in a health domain that are not only accurate but also just and developed for full human interaction, we need to consider and define in detail our understanding of what health is. Mistakes, biases, and inequalities will inevitably arise, but they might be easier to spot and resolve thanks to the conceptual transparency afforded by justificatory explanations. A non-technical intervention like the one we are proposing here might aid the critical work that is required to properly evaluate intrinsically fraught systems going beyond examining datasets and code.

We should avoid the oracular approach to artificial intelligence, in which an algorithm generates “objective values” based on “pure data”, and humans are no longer necessary. Humans (from policymakers to civil society to individuals) need to have access to an explanation that offers an account of how the algorithm works in terms they can understand. We need to consider how any computer-based intervention in medicine starts with a specific understanding of health, which is linked to values that are very difficult to turn into raw data. Therefore, we need to change our paradigm and create systems based on other models where humans and algorithms interact, instead of just the algorithm stating like an oracle what the human must do. In this view, for instance, a diagnosis is made by a human-assisted by the machine, and a key element in such collaboration is how the computer system enhances human decision-making, but does not replace it with something whose justification cannot be fully comprehended or is outright unavailable. Such systems need to include in their documentation the key concepts (in our examples, the justificatory explanations of “health”) that underpinned its design and development, so the human users and stakeholders can have a proper understanding of what the engineering team aimed at and what the implications of its use might be.

We want to end by considering one objection to our proposal, which would be to reiterate that most model and software developers are technically oriented people who are usually not trained in philosophy or the social sciences. The thrust of the objection is that they would have difficulty with or would be unable to produce fully blown explanatory justifications or precise descriptions of the key concepts that underpin the system they are designing or have designed. We would reply that all we are asking is that *they externalise concepts they must already possess and are already guiding their decision-making either explicitly or implicitly*. We find it reasonable to expect that the creators of high-impact systems in areas such as health, security, or education, to name a few, must be able to explain to others what they understand by the *thick*, value-laden and fraught concepts on which their work is ultimately based. We posit that it is not unreasonable to expect from them and from other relevant decision-makers involved in the design of AI systems to provide explanations in terms that can be understood and evaluated by the public. We would also contend that the higher the potential impact of a system in our lives, the more explicit and sophisticated the understanding of these key concepts needs to be and the more developers and decision-makers need to be aware of alternative conceptual understandings. Now, we have nothing like the space we would need to discuss *how* this could be done, but it is clear that it might be necessary for AI teams to work with experts from other domains such as philosophy, anthropology, sociology, and the humanities (for integrating ethicists in the process, see Van Wynsberghe and Robbins 2014). Our argument is *that* it needs to be done. If we really want transparency, accountability, and explainability, we need to go all the way beyond code and data to illuminate the other black box that resides in the minds of all of those who design and develop AI systems. Providing justificatory explanations might be a good start.

## Footnotes

1 Searle (2001: pp. 110–111) uses this same term, but our understanding of “justificatory reasons” is somewhat different and more lenient than his.

2 We make no claims about the truth value of “everyday” understandings. Our goal is simply to emphasise its often tacit and intuitive nature, which may or may not be correctly aligned with scholarly theories and scientific evidence. Because we take no issue with this discussion, we prefer not to use the terms “lay theories” (or “folk theories”), which are often contrasted with scientific theories and have connotations we prefer to avoid.
